# Supplementary material for: Impact on Bile Acid Concentrations by Alveolar Echinococcosis and Treatment with Albendazole in Mice
Source: Metabolites. 2021 Jul 6;11(7):442. doi: 10.3390/metabo11070442 (PMC8307106; doi:10.3390/metabo11070442)
Supplement: Supplementary file 1 [file metabolites-11-00442-s001.zip › metabolites-1280706-supplementary.pdf]

Supplementary file

Impact on Bile Acid Concentrations by Alveolar Echinococcosis and Treatment with Albendazole in Mice

Cristina Gómez [1], Fadi Jebbawi [1], Michael Weingartner [1], Junhua Wang [2,3], Simon Stücheli [1], Bruno Stieger [4], Bruno Gottstein [2,3], Guido Beldi [5], Britta Lundström-Stadelmann [2] and Alex Odermatt [1,*]

1 Division of Molecular and Systems Toxicology, Department of Pharmaceutical Sciences, University of Basel, Klingelbergstrasse 50, 4056 Basel, Switzerland; cristina.gomezcastella@unibas.ch (C.G.); fadi.jebbawi@unibas.ch (F.J.); michael.weingartner@unibas.ch (M.W.); simon.stuecheli@unibas.ch (S.S.)
2 Department of Infectious Diseases and Pathobiology, Vetsuisse Faculty, Institute of Parasitology, University of Berne, Längassstrasse 122, 3012, Berne, Switzerland; junhua.wang@ifik.unibe.ch (J.W.); bruno.gottstein@ifik.unibe.ch (B.G.); britta.lundstroem@vetsuisse.unibe.ch (B.L.-S.)
3 Faculty of Medicine, Institute for Infectious Diseases, University of Berne, Friedbühlstrasse 51, 3010, Berne, Switzerland
4 Department of Clinical Pharmacology and Toxicology, University Hospital Zurich, University of Zurich, 8091 Zurich, Switzerland; bstieger@ethz.ch
5 Department of Visceral Surgery and Medicine, University Hospital of Berne, Freiburgstrasse, 3010 Berne, Switzerland; Guido.Beldi@insel.ch
* Correspondence: alex.odermatt@unibas.ch

Table S1: Optimized multiple reaction monitoring (MRM) transitions used as quantifiers and qualifiers, electrospray ionization (ESI) mode (positive (+) or negative (-)), and collision energies (CE).

Compound	Abbreviation	ESI	RT (min)	Transition	CE (V)	Transition	CE (V)
Tauro-ω-muricholic acid	TωMCA	+	2.82	480.3 > 126	24	480.3 > 462.1	8
Tauro-α-muricholic acid	TαMCA	+	3.07	480.3 > 126	24	480.3 > 462.1	8
Tauro-β-muricholic acid	TβMCA	+	3.30	480.3 > 126	24	480.3 > 462.2	8
Tauro-ursodeoxycholic acid	TUDCA	+	5.20	464.2 > 126	28	464.2 > 464.2	0
Tauro-ursodeoxycholic acid	TUDCA	-	5.20	498.2 > 124.2	47	-	-
Tauro-cholic acid	TCA	+	5.45	480.3 > 461.9	8	480.3 > 126	24
Tauro-cholic acid	TCA	-	5.45	514.2 > 124.2	46	-	-
Tauro-7-oxolithocholic acid	T7oxoLCA	+	5.84	480.3 > 126	24	480.3 > 461.9	8
ω-muricholic acid	ωMCA	+	6.29	373.3 > 159.1	20	373.3 > 355.2	15
Glyco-ursodeoxycholic acid	GUDCA	-	6.43	448.2 > 74	37	448.2 > 386.1	35
Glyco-ursodeoxycholic acid	GUDCA	-	6.43	448.2 > 404.1	40	-	-
α-muricholic acid	αMCA	+	6.50	373.3 > 355.2	15	373.3 > 105.1	
Glyco-cholic acid	GCA	-	6.63	464.4 >74	37	464.2 > 402.1	25
7-oxo-deoxycholic acid	7oxoDCA	+	6.81	371.3 > 353.2	8	371.3 > 335.2	12
β-muricholic acid	βMCA	+	6.85	391.3 > 355.2	16	373.3 > 355.2	8
β-muricholic acid	βMCA	+	6.85	373.3 > 105.1	58	-	-
Glyco-7-oxo-lithocholic acid	G7oxoLCA	-	7.63	446.2 > 74	37	446.2 > 330.1	49
Tauro-chenodeoxycholic acid	TCDCA	+	7.91	464.2 > 126	28	464.2 > 464.2	0
Tauro-chenodeoxycholic acid	TCDCA	-	7.91	498.2 > 124.2	45	-	-
γ-muricholic acid	γMCA	+	8.14	373.3 > 355	16	391.3 > 355.2	10
Tauro-deoxycholic acid	TDCA	-	8.79	498.2 > 124.2	45	-	-

Tauro-deoxycholic acid	TDCA	+	8.79	464.2 > 126	28	464.2 > 464.2	0
Allo-cholic acid	AlloCA	+	9.44	373.3 > 355.2	12	373.3 > 159.1	20
Cholic Acid	CA	+	9.81	373.3 > 355.2	8	373.3 > 159.1	20
Ursodeoxycholic acid	UDCA	+	9.92	357.2 > 95	35	357.2 > 80.9	50
Allo-3β-deoxycholic acid	Allo3βDCA	-	10.10	391.3 > 345.1	40	391.3 > 342.8	40
Hyodeoxycholic acid	HDCA	+	10.34	357.2 > 95.1	40	357.3> 104.8	50
Glyco-chenodeoxycholic acid	GCDCA	-	10.58	448.2 > 74	30	448.2 > 404.1	32
Glyco-deoxycholic acid	GDCA	-	11.43	448.2 > 74	30	448.2 > 404.1	32
Glyco-deoxycholic acid	GDCA	-	11.43	448.2 > 386.1	35	-	-
6,7-dioxo-lithocholic acid	6,7dioxoLCA	+	11.71	405.3 > 351.1	12	405.3 > 333.1	16
7-oxolithocholic acid	7oxoLCA	+	11.80	373.3 > 355.1	8	373.3 > 105.1	58
Allo-12β-deoxycholic acid	Allo12βDCA	-	11.94	391.3 > 345.1	36	391.3 > 327.3	40
12-oxo-lithocholic acid	12oxoLCA	+	12.25	391.3 > 145.1	32	391.3 > 309.3	20
Tauro-lithocholic acid	TLCA	-	13.11	482.2 > 80	56	482.2 > 107.2	50
Tauro-lithocholic acid	TLCA	-	13.11	482.2 > 124.2	45	-	-
Chenodeoxycholic acid	CDCA	+	13.76	357.2 > 104.9	50	357.2 > 81.1	48
Allo-deoxycholic acid	AlloDCA	-	13.91	391.3 > 345	36	391.3 > 327.1	40
Allo-deoxycholic acid	AlloDCA	+	13.91	357.2 > 104.9	50	357.2 > 81.1	48
Deoxycholic acid	DCA	-	14.14	391.3 > 345	36	391.3 > 327.1	40
Deoxycholic acid	DCA	+	14.14	357.2 > 104.9	50	357.2 > 81.1	48
Glyco-lithocholic acid	GLCA	-	14.59	432.2 > 74	41	432.2 > 388.1	37
Allo-3β-lithocholic acid	Allo3βLCA	+	15.56	359.3 > 135.1	25	359.3 > 95.1	32
Allo-lithocholic acid	AlloLCA	+	17.08	359.3 > 135.1	25	359.3 > 95.1	32
Lithocholic acid	LCA	+	17.19	359.3 > 135.1	24	359.3 > 95.1	32
3-oxo-lithocholic acid	3oxoLCA	+	17.43	357.3 > 80.9	48	357.3 > 95.1	40
**Internal Standards**							
d4-Glyco-ursodeoxycholic acid	d4-GUDCA	-	6.40	452.2 >74	37	-	-
d4-Glyco-cholic acid	d4-GCA	-	6.61	468.2 > 74	49	-	-
d4-Cholic acid	d4-CA	+	9.79	377.3 > 359.2	8	-	-
d4-Ursodeoxycholic acid	d4-UDCA	+	10.02	361.2 > 95.1	40	-	-
d4-Glyco-chenodeoxycholic acid	d4-GCDCA	-	10.55	452.2 > 74	37	-	-
d4-Chenodeoxycholic acid	d4-CDCA	+	13.70	361.3 > 95.1	40	-	-
d4-Deoxycholic acid	d4-DCA	+	14.16	361.3 > 95.1	40	-	-
d4-Lithocholic acid	d4-LCA	+	17.05	363.3 > 139.1	24	-	-

Table S2: Oligonucleotide primers for mRNA quantification by qPCR.

Gene	Forward	Reverse
Bsep	TGGTAGAGAAGAGGCGACAAT	TGAGGTAGCCATGTCCAGAA
Ntcp	GCCACACTATGTACCCTACGTC	TTTAGTCGGAAGAGAGCAGAGA
Mrp4	CATCAAGTCCAGGGAAAAGGTTG	GAGGGCCGAGATGAGGGAG
Mrp2	GCTTAGTTCAAGTCTATGGAGT	TCCGGCCGATACCGCACTTGATA
Cyp7a1	CTTGAGGATGGTTCCTATAAC	TTAAAAGTCAAAGGGTCTGG
Cyp27a1	AAAGCTGTGATTAAGGAGAC	CAAACTGTGTATTCTTGGGG
Oatp1b2	CAACCTGACTGGTTTTCTATG	AAGTGAAGGATCCAATGAAG
Slc51a (Ostα)	CTTGACCCCAGGTACACAGC	ATGGGGCAAAGGGTGTTCTT
Slc51b (Ostβ)	AGATGCGGCTCCTTGGAATTA	TGGCAGAAAGACAAGTGATG

Oatp1a1	TAGCTTGCCTCCAGTATGCCTT	ACAGGCCAAATGCTATGTATGC
Akr1d1	GAAAAGATAGCAGAAGGGAAGGT	GGGACATGCTCTGTATTCCATAA
Actb	ACCCTGTGCTGCTCACCGA	CTGGATGGCTACGTACATGGCT

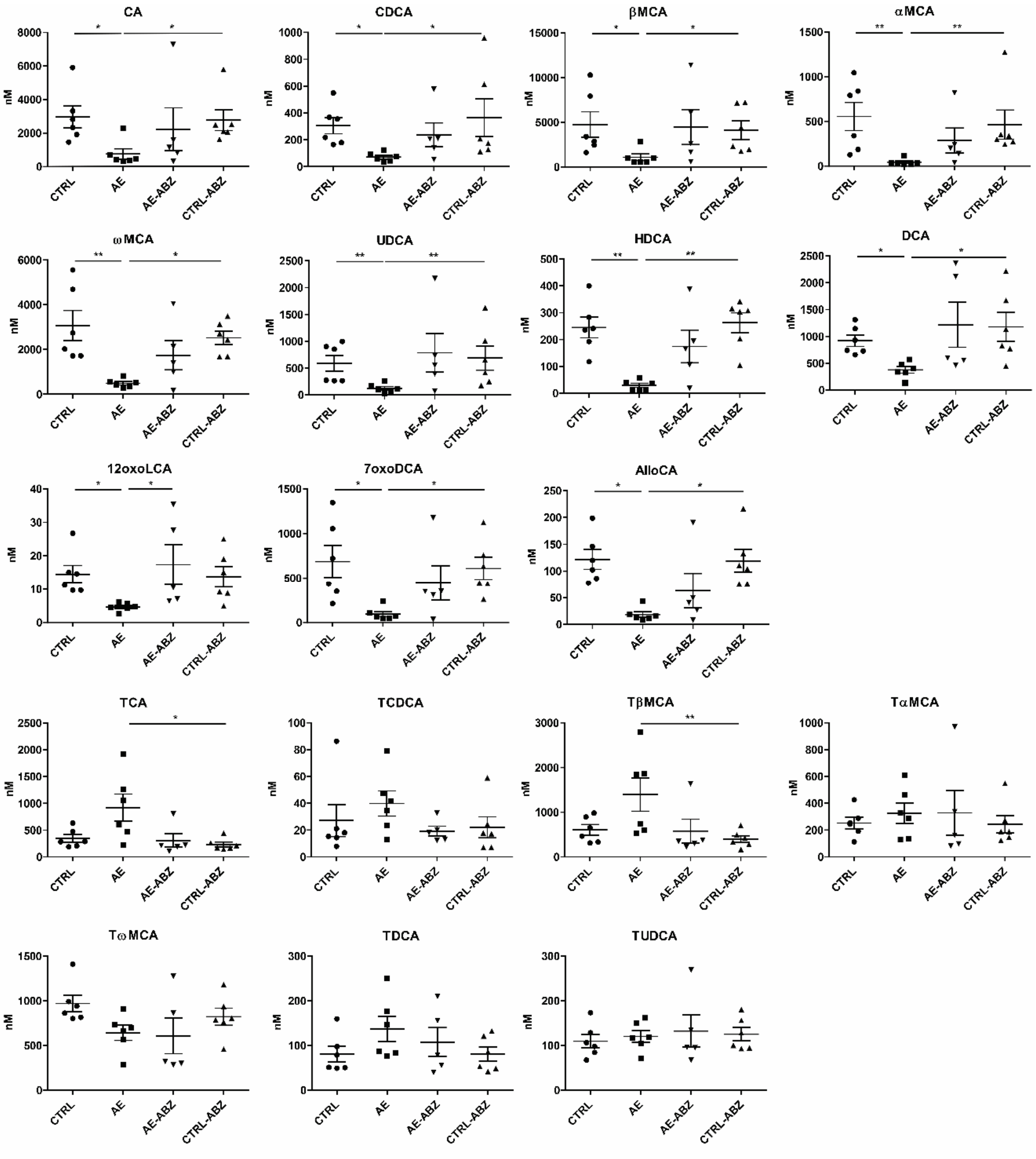

Figure S1. Concentrations of individual bile acids quantified in serum of mice from the four treatment groups. Non-infected control (CTRL, n=6), *E. multilocularis* infected (AE, n=6), *E. multilocularis* infected and ABZ treated (AE-ABZ, n=5, one outlier with aberrant concentrations was removed), and non-infected and ABZ treated control mice (CTRL-ABZ, n=6). Values are expressed as mean ± SD (nM). * $p < 0.05$ and ** $p < 0.01$.

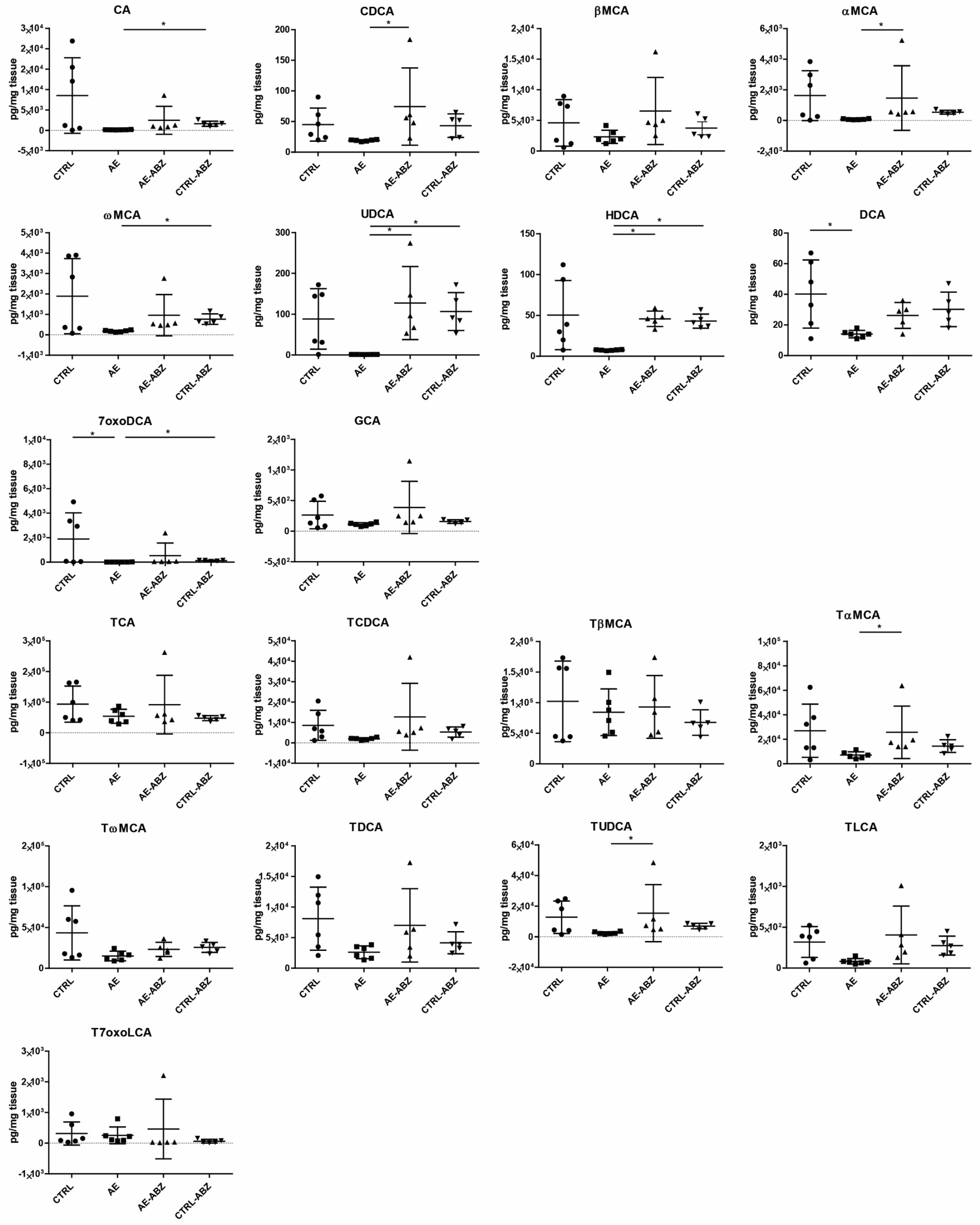

Figure S2. Concentrations of individual bile acids quantified in liver tissue of mice from the four treatment groups. Non-infected control (CTRL, n=6), *E. multilocularis* infected (AE, n=6), *E. multilocularis* infected and ABZ treated (AE-ABZ, n=5, one outlier with aberrant concentrations was removed), and non-infected and ABZ treated control mice (CTRL-ABZ, n=5, one outlier with aberrant concentrations was removed). Values are expressed as mean ± SD (pg/mg tissue). * $p < 0.05$ and ** $p < 0.01$.

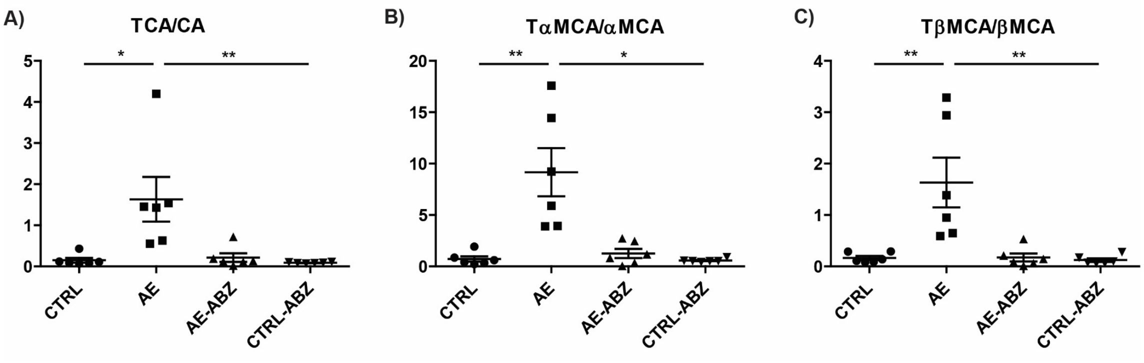

Figure S3: Increased ratios of taurine-conjugated to unconjugated bile acids in serum of AE mice. The ratios of the serum concentrations of the taurine-conjugated cholic acid (A), α-muricholic acid (B) and β-muricholic acid (C) to the respective unconjugated bile acid were determined in the four different treatment groups. Non-infected control (CTRL, n=6), *E. multilocularis* infected (AE, n=6), *E. multilocularis* infected and ABZ treated (AE-ABZ, n=5, one outlier with aberrant concentrations was removed), and non-infected and ABZ treated control mice (CTRL-ABZ, n=6). Values are expressed as mean ± SD. * $p < 0.05$ and ** $p < 0.01$.

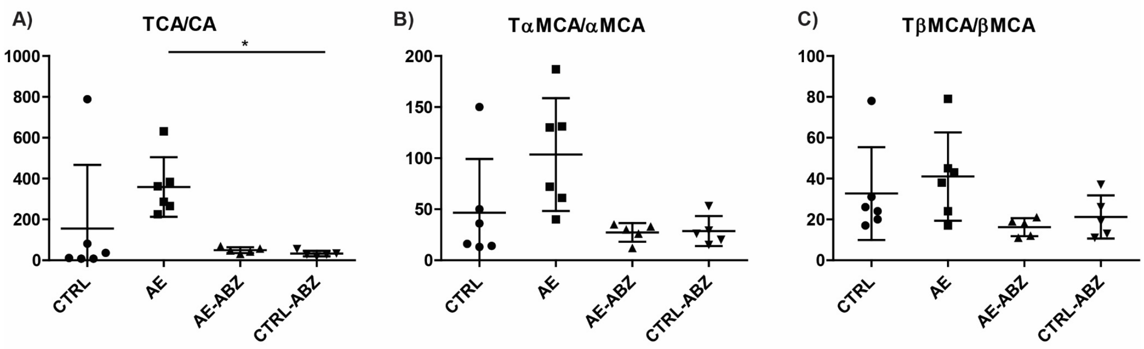

Figure S4: Ratios of taurine-conjugated to unconjugated bile acids in liver tissues of AE mice. The ratios of the liver tissue concentrations of the taurine-conjugated cholic acid (A), α-muricholic acid (B) and β-muricholic acid (C) to the respective unconjugated bile acid were determined in the four different treatment groups in liver tissue. Non-infected control (CTRL, n=6), *E. multilocularis* infected (AE, n=6), *E. multilocularis* infected and ABZ treated (AE-ABZ, n=5, one outlier with aberrant concentrations was removed), and non-infected and ABZ treated control mice (CTRL-ABZ, n=5, one outlier with aberrant concentrations was removed). Values are expressed as mean ± SD. * $p < 0.05$.

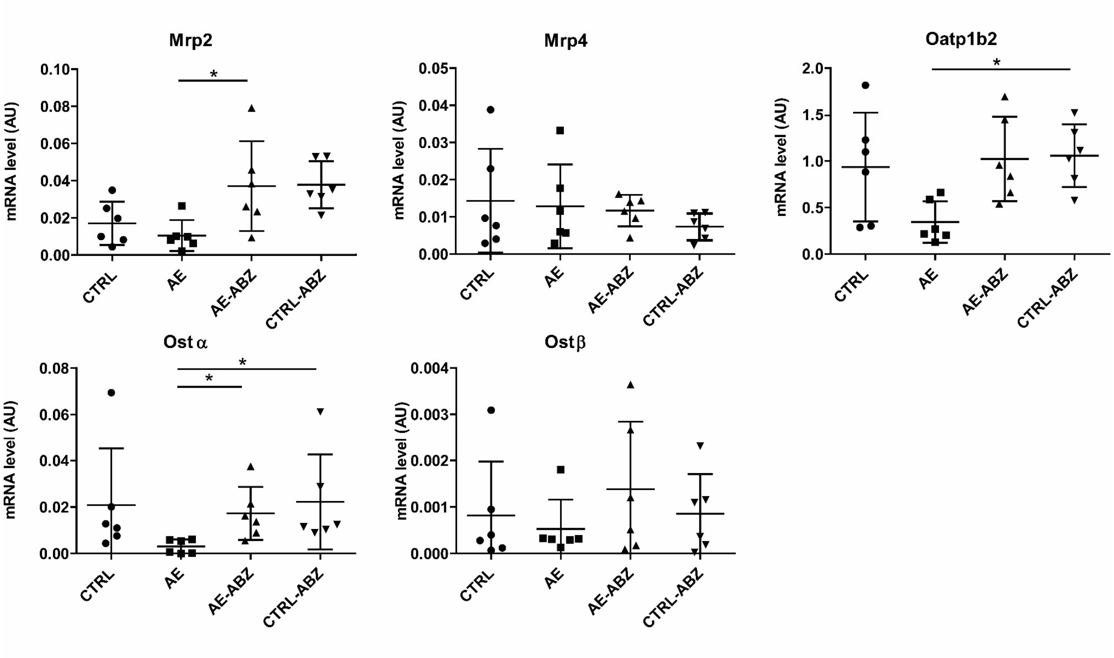

Figure S5. Effect of AE on mRNA expression levels of additional bile acid transporters. The mRNA levels of Mrp2, Mrp4, Ostα, Ostβ, and Oatp1b2 were determined in liver tissues of non-infected control (CTRL, n=6), *E. multilocularis* infected (AE, n=6), *E. multilocularis* infected and ABZ treated (AE-ABZ, n=6), and non-infected and ABZ treated control mice (CTRL-ABZ, n=6). Values are expressed as mean ± SD. * $p < 0.05$.
